# Statin use and its association with decreased risk of esophageal squamous cell carcinoma in betel nut chewers

**DOI:** 10.1111/1759-7714.15009

**Published:** 2023-07-03

**Authors:** Chih‐Lang Lin, Wan‐Ming Chen, Ben‐Chang Shia, Szu‐Yuan Wu

**Affiliations:** ^1^ Liver Research Center, Department of Gastroenterology and Hepatology Keelung Chang Gung Memorial Hospital Keelung Taiwan; ^2^ Community Medicine Research Center Keelung Chang Gung Memorial Hospital Keelung Taiwan; ^3^ College of Medicine Chang Gung University Taiwan; ^4^ Graduate Institute of Business Administration, College of Management Fu Jen Catholic University Taipei Taiwan; ^5^ Artificial Intelligence Development Center Fu Jen Catholic University Taipei Taiwan; ^6^ Department of Food Nutrition and Health Biotechnology, College of Medical and Health Science Asia University Taichung Taiwan; ^7^ Division of Radiation Oncology, Lo‐Hsu Medical Foundation Lotung Poh‐Ai Hospital Yilan Taiwan; ^8^ Big Data Center, Lo‐Hsu Medical Foundation Lotung Poh‐Ai Hospital Yilan Taiwan; ^9^ Department of Healthcare Administration, College of Medical and Health Science Asia University Taichung Taiwan; ^10^ Cancer Center, Lo‐Hsu Medical Foundation Lotung Poh‐Ai Hospital Yilan Taiwan; ^11^ Centers for Regional Anesthesia and Pain Medicine, Taipei Municipal Wan Fang Hospital Taipei Medical University Taipei Taiwan; ^12^ Department of Management, College of Management Fo Guang University Yilan Taiwan

**Keywords:** betel nut chewers, dose–response relationship, ESCC, incidence, statin

## Abstract

**Background:**

Betel nut chewing involves the chewing of areca nuts or betel quid (areca nuts wrapped in betel leaves), which is associated with an increased risk of esophageal squamous cell carcinoma (ESCC). Statins have anticancer properties. We investigated the association between statin use and ESCC risk in betel nut chewers.

**Methods:**

The study included 105 387 betel nut chewers matched statin users and nonusers. Statin use was defined as the use of ≥28 cumulative defined daily doses (cDDDs) of statin. The primary outcome was incidence of ESCC.

**Results:**

The incidence rate of ESCC was significantly lower in statin users than in nonusers (2.03 vs. 3.02 per 100 000 person‐years). Statin users had a lower incidence rate ratio of 0.66 for ESCC (95% confidence interval [CI]: 0.43–0.85) relative to nonusers. After potential confounders were adjusted for, statin use was determined to be associated with a reduced risk of ESCC (adjusted hazard ratio [aHR], 0.68; 95% CI: 0.51–0.91). A dose–response relationship was observed between statin use and ESCC risk; the aHRs for statin use at 28–182 cDDDs, 183–488 cDDDs, 489–1043 cDDDs, and > 1043 cDDDs were 0.92, 0.89, 0.66, and 0.64, respectively.

**Conclusion:**

Statin use was revealed to be associated with a reduced risk of ESCC in betel nut chewers.

## INTRODUCTION

Betel nut chewing is a widespread practice in Taiwan and was first introduced to the majority Han population by the native indigenous peoples of Taiwan.^1^ Estimates indicate that more than NT$100 billion is spent annually on this product, which is colloquially known as “Taiwanese chewing gum.”^2^ The high consumption of betel nut, cigarettes, and alcohol in Taiwan has contributed to its high incidence of head and neck cancer.^3^ The prevalence of betel nut chewing in Taiwan (lifetime prevalence of 10%)^4^ has caused it to become a major public health concern. Betel nut chewing involves the chewing of areca nuts or betel quid (areca nuts wrapped in betel leaves), which is associated with an increased risk of several types of cancer,^5^ including esophageal squamous cell carcinoma (ESCC).^6^, ^7^ The exact mechanism through which betel nut chewing increases the risk of ESCC is not fully understood, but it is speculated to involve the release of copper, which leads to collagen synthesis by fibroblasts.^8^ This phenomenon is also associated with a younger age of diagnosis, poor chemotherapy response, poor radiotherapy response, and shorter overall survival in patients with ESCC.^9^ Given the high prevalence of betel nut chewing in the Taiwanese population, the habit is a particularly relevant risk factor for ESCC in Taiwan. The overall survival rate for ESCC is poor both globally and in Taiwan.^10^, ^11^, ^12^, ^13^ The mean age of diagnosis of ESCC in Taiwan is approximately 50 years old,^10^, ^11^, ^12^, ^13^ and patients with ESCC are often economically active individuals and the main breadwinners in their families. Discovering effective protective medications against ESCC is a valuable and crucial goal in the context of a high population of betel nut chewers.

Statins are a class of drugs that are commonly used to reduce cholesterol levels and the risk of cardiovascular disease.^14^ Studies have indicated that statins have anticancer properties.^15^, ^16^ Several studies have reported an association between statin use and a reduced risk of various types of cancer, including esophageal, gastric, colorectal, liver, and lung cancer.^17^ Although the mechanisms underlying the anticancer effects of statins remain unclear, several explanations have been proposed. The first explanation is that statins inhibit cancer cell growth and spread by reducing proteolysis and having antiproliferative, antiangiogenic, proapoptotic, and immunomodulatory effects.^18^, ^19^ The second explanation is that statins modulate fibrosis progression, which is an underlying mechanism that contributes to the development of cancer.^20^ Studies have also reported that statins have strong antifibrosis, anti‐inflammatory, and immunomodulatory effects.^21^ Statins reduce the activity of inflammatory signaling pathways,^22^, ^23^ which is associated with an increased cancer risk. In addition, several studies have indicated that statin use is associated with a reduced esophageal cancer risk, especially in patients with Barrett's esophagus.^24^, ^25^, ^26^

We conducted a long‐term, head‐to‐head comparative national cohort study by employing propensity score matching (PSM) to understand the association between statin use and the risk of ESCC in betel nut chewers who are at high risk of ESCC and have poor overall survival, particularly in relation to their betel nut chewing habits. Additionally, we verified whether a dose–response relationship was present. The present study is the first to investigate the relationship between statin use and ESCC risk in betel nut chewers, and it provides valuable insights that can guide future research in this area.

## METHODS

### Study population

We conducted a population‐based cohort study using data from the Taiwan National Health Insurance (NHI) Research Database (NHIRD) for the period from 2008 to 2018. The NHIRD stores comprehensive medical claims data on all NHI beneficiaries, including their diagnoses, procedures, drug prescriptions, demographics, and enrollment profiles, all of which are encrypted using unique patient identifiers.^27^, ^28^, ^29^, ^30^, ^31^, ^32^ Because the NHIRD is linked to the death registry, the vital status and cause of death of each included patient could be determined. The NHIRD is a valuable resource for population‐based research because it covers the entire NHI‐insured population of Taiwan, which represents more than 99% of the Taiwanese population.^28^, ^29^, ^30^, ^31^, ^32^ In Taiwan, the Health Promotion Administration of the Ministry of Health and Welfare initiated an oral cancer screening program in 2004.^33^ The population determined to be at the highest risk of developing oral cancer was determined to be that with a betel nut chewing habit, and patients with this habit were identified through a link between the National Oral Cancer Screening database and the NHIRD. Therefore, we included patients who were betel nut chewers and enrolled in the linked NHIRD and National Oral Cancer Screening database.

Our study included patients who were betel nut chewers, aged ≥20 years, and enrolled in the NHIRD and the National Oral Cancer Screening database; patients with missing age data were excluded. Statin use was defined as the use of ≥28 cumulative defined daily doses (cDDDs) of statin. The index date was the date on which a patient's statin use reached 28 cDDDs. The observation period for each patient began on the index date and continued until the patient was diagnosed with ESCC or until the end of the study period (December 31, 2021). Patients who died during the observation period were excluded to ensure that no competing risk of mortality was present between the case and control groups. The patients who were prescribed ≥28 cDDDs and <28 cDDDs of statin during the follow‐up period formed the case group (statin users) and the control group (statin nonusers), respectively. The follow‐up duration was defined as 1 year after the date of initial statin use or cohort entry. The present study is the first to investigate the association between statin use and ESCC risk, and it provides valuable information to clarify the risk of ESCC in the general population.

Patients were excluded if (1) they were given a diagnosis of ESCC within 1 year of the index date, (2) they had missing data pertaining to their sex or age or were aged <20 years, (3) they had a follow‐up duration of <1 year, or (4) they were given a diagnosis of any other type of cancer within 1 year of the cohort entry date (this criterion prevented ESCC‐related metastases from influencing the results). These criteria were implemented to ensure that our results would accurately reflect the association between statin use and ESCC risk.

The study protocols were reviewed and approved by the Institutional Review Board of Tzu‐Chi Medical Foundation (IRB109‐015‐B).

### Study covariates

To control for potential confounding factors, we included several covariates in our analysis. The study participants were divided into four age groups (20–50, 51–60, 61–70, and ≥71 years) on the basis of their age on the index date. The index date for a statin user was defined as the date on which their statin use reached 28 cDDDs. For the matched statin nonusers, we used the variable data collected on the index date. To prevent repeated adjustments in our multivariate analysis, we excluded repeated comorbidities from our Charlson Comorbidity Index (CCI) calculations. We identified comorbidity onset within 1 year of the index date by using the *International Classification of Diseases* codes from either the main inpatient diagnosis or those from ≥2 outpatient visits within 1 year. These codes were from either the Ninth Revision, Clinical Modification (ICD‐9‐CM) or the Tenth Revision, Clinical Modification (ICD‐10‐CM).

### Statin exposure

Statin use was defined as the use of ≥28 cDDDs of a statin.^29^, ^34^ Data on the drug type, dosage, administration route, prescription date, and total number of pills dispensed by a pharmacy were collected. Because statins could have been used in nonconsecutive years during the study period and the patients could have changed their drug use patterns over time, we treated statin use as a time‐varying covariate in the Cox model.^35^ The cumulative dose of statins was calculated by multiplying the number of pills dispensed for a prescribed dose and dividing the result by the recorded days' supply. The defined daily dose (DDD) of statins, as established by the World Health Organization, was used to express dosage. DDD is the average maintenance dose per day for a drug that is used for its main indication in adults, and cDDDs were calculated as the sum of the DDDs. Statin nonuse was defined as the absence of statin use or statin use amounting to <28 cDDDs (excluding occasional statin use), whereas statin use was defined as statin use amounting to ≥28 cDDDs. The included patients were divided into four subgroups on the basis of cDDD quartiles (Tables 1 and 3).

**TABLE 1:** Baseline characteristics of statin users and nonusers among betel nut chewing patients after propensity score matching.

	Statin nonusers		Statin users		*ASMD*
	*n* = 105 387		*n* = 105 387		
	*n*	%	*n*	%	
Age (mean ± SD), years	53.68 ± 19.88		58.00 ± 12.55		0.000
Age, median (IQR, Q1, Q3), years	57.00 (46.00, 67.00)		58.00 (50.00, 67.00)		
Age group, years					0.000
≤50	28 625	27.16%	28 625	27.16%	
51–60	33 216	31.52%	33 216	31.52%	
61–70	25 356	24.06%	25 356	24.06%	
>70	18 190	17.26%	18 190	17.26%	
Sex					0.000
Female	54 649	51.86%	54 649	51.86%	
Male	50 738	48.14%	50 738	48.14%	
Income (NTD)					0.001
Low income	839	0.80%	867	0.82%	
10 000–19 999	28 454	27.00%	28 444	26.99%	
≤ 20 000	29 781	28.26%	29 843	28.32%	
20 001–30 000	24 077	22.85%	23 898	22.68%	
30 001–45 000	13 826	13.12%	13 898	13.19%	
> 45 000	8410	7.098%	8437	8.01%	
Urbanization level					0.002
Rural	31 118	29.53%	28 953	27.47%	
Urban	74 268	70.47%	76 433	72.53%	
Cigarette smoking habit	9833	9.33%	9801	9.30%	0.001
Alcohol‐related diseases	2086	1.98%	2101	1.99%	0.001
Comorbidities					
Diabetes	39 650	37.62%	39 652	37.63%	0.000
Hypertension	63 515	60.27%	63 519	60.27%	0.000
Chronic obstructive pulmonary disease	20 238	19.20%	20 241	19.21%	0.000
Gastroesophageal reflux disease	4974	4.72%	4979	4.72%	0.000
Barrett's esophagus	1172	1.11%	1179	1.12%	0.000
Obesity	2034	1.93%	2035	1.93%	0.000
Achalasia	83	0.08%	84	0.08%	0.000
Tylosis (Howel–Evans syndrome)	744	0.71%	731	0.69%	0.001
Plummer–Vinson syndrome	60	0.06%	61	0.06%	0.000
Medication use					
Aspirin	57 722	54.77%	57 730	54.78%	0.001
Metformin	41 719	39.59%	41 722	39.59%	0.000
PPI	15 614	14.82%	15 618	14.82%	0.000
CCI score					
Mean (SD)	0.76 ± 0.88		0.78 ± 1.13		0.001
Median (IQR, Q1–Q3)	0.00 (0.00, 0.00)		0.00 (0.00, 0.00)		
CCI score					0.001
0	84 653	80.33%	84 623	80.30%	
≥1	20 734	19.67%	20 750	19.70%	
cDDD of statins					
Nonuse	105 387	100.00%	0	0.00%	
Q1	0	0.00%	26 526	25.17%	
Q2	0	0.00%	26 211	24.87%	
Q3	0	0.00%	26 307	24.96%	
Q4	0	0.00%	26 343	25.00%	
					*p* value
Mean (± SD) follow‐up, years	6.73 ± 4.09		6.92 ± 4.09		0.649
Median (IQR, Q1, Q3) follow‐up, years	6.12 (3.31, 9.62)		6.39 (3.53, 9.79)		0.347
Primary outcome					
ESCC					<0.001
No	105 073	99.80%	105 266	99.79%	
Yes	314	0.30%	121	0.11%	

Abbreviations: ASMD, absolute standardized mean difference; CCI, Charlson Comorbidity Index; cDDD, cumulative defined daily dose; DDD, defined daily dose; ESCC, esophageal squamous cell carcinoma; IQR, interquartile range; *n*, number; NTD, New Taiwan dollars; PPI, proton pump inhibitor; PSM, propensity score matching; SD, standard deviation.

### PSM and covariates

We used a time‐varying Cox proportional hazards model to analyze the relationship between statin use and the onset of ESCC after controlling for potential confounders. To minimize the effect of confounding factors during the comparison of the ESCC risk of statin users and nonusers, we matched the patients on the basis of their propensity scores. The variables used for matching were age, sex, income level, urbanization level, cigarette smoking habit, alcohol‐related diseases, comorbidities (diabetes, hypertension, chronic obstructive pulmonary disease, gastroesophageal reflux disease, Barrett's esophagus, obesity, achalasia, tylosis [Howel‐Evans syndrome], Plummer–Vinson syndrome), medication use (aspirin, metformin, and proton pump inhibitors [PPIs]), and CCI score. Comorbidities were identified using the *International Classification of Diseases, Ninth Revision*; *Clinical Modification or International Classification of Diseases, Tenth Revision*; and the clinical modification codes from one inpatient visit or ≥2 outpatient visits within 1 year of a main diagnosis. We excluded repeat comorbidities from our CCI calculations to prevent repeated adjustments in our multivariate analysis.

In the present study, continuous variables are presented as means with standard deviations or medians with first and third quartiles. To minimize the differences between the patient groups, we employed the greedy method as a matching technique; specifically, PSM was conducted with a caliper width of 0.2 to match the patients at a ratio of 1:1.^36^ This method involves selecting controls with identical background covariates that must be controlled for.

#### Primary endpoints

The primary outcome of this study was the occurrence of ESCC, which was confirmed by checking the certification records in the Registry for Catastrophic Illness Patients.^37^

#### Statistical analysis

We collected information on patient characteristics, namely the included patients' age, sex, comorbidities, and statin dosage. Age was divided into 10‐year intervals, and the baseline characteristics of statin users and nonusers were compared by performing chi‐square tests for categorical variables, *t* tests for continuous variables, and Wilcoxon rank‐sum tests for median values. The cohort entry date was set as the baseline. To assess the association between statin use and risk of ESCC, we calculated incidence rates (IRs) and incidence rate ratios (IRRs) and estimated adjusted hazard ratios (aHRs) with 95% confidence intervals (CIs) by employing Cox regression models and adjusting for age, sex, income level, urbanization level, cigarette smoking habit, alcohol‐related diseases, comorbidities, medication use, and CCI score. The cumulative incidence of ESCC of the stain users and nonusers was estimated using the Kaplan–Meier method and compared using the log‐rank test.

All statistical analyses were performed using SAS for Windows (version 9.4; SAS Institute, Cary, NC, USA), and a two‐sided *p*‐value of <0.05 was regarded as statistically significant.

## RESULTS

### Baseline characteristics of study population

We analyzed the data of 210 774 individuals who were enrolled (in the aforementioned databases) between 2008 and 2018. The final follow‐up date was December 31, 2020. To compare the data of the statin user and statin nonuser groups, we performed individual 1:1 matching, and each group comprised 105 387 patients. The age distribution of the two groups was similar (Table 1). Our PSM results revealed that the statin user and statin nonuser groups were comparable with respect to the variables of sex, income level, urbanization level, cigarette smoking habit, alcohol‐related diseases, comorbidities, medication use, and CCI score.

### Association of comorbidities and concurrent medications with ESCC risk

Table 2 presents the association of ESCC risk with concurrent medications and comorbidities in our study cohort. The risk of ESCC increased with age (patients aged 20–50 years were the reference group). Men had a higher risk of ESCC relative to women (aHR, 2.78; 95% CI: 2.23–3.46). Cigarette smokers had a higher risk of ESCC relative to nonsmokers (aHR, 1.21; 95% CI: 1.12–1.59), and patients with alcohol‐related diseases had a higher risk of ESCC relative to those without such diseases (aHR: 6.42; 95% CI: 4.34–9.47).

**TABLE 2:** Time‐varying Cox regression model of association of comorbidities and concurrent medications with esophageal squamous cell carcinoma risk in patients with betel nut chewing habits.

	Crude HR	(95% CI)	*p*	aHR^a^	(95% CI)	*p*
Statin use (ref. nonuser)						
Statin user	0.71	(0.55, 0.91)	0.006	0.68	(0.51, 0.91)	0.010
Sex (ref. female)						
Male	2.34	(1.89, 2.89)	<0.001	2.78	(2.23, 3.46)	<0.001
Age group, years (ref. 18, 19, 20, 21, 22, 23, 24, 25, 26, 27, 28, 29, 30, 31, 32, 33, 34, 35, 36, 37, 38, 39, 40, 41, 42, 43, 44, 45, 46, 47, 48, 49, 50)						
51–60	2.11	(1.51, 2.96)	<0.001	2.76	(1.96, 3.89)	<0.001
61–70	2.59	(1.85, 3.64)	<0.001	3.29	(2.31, 4.7)	<0.001
>70	4.49	(3.2, 6.29)	<0.001	5.67	(3.92, 8.2)	<0.001
Income (Ref. Low income, NTD)						
10 000–19 999	1.00	(0.77, 1.31)	0.9787	1.11	(0.85, 1.45)	0.448
≤ 20 000	0.89	(0.71, 1.12)	0.3204	1.06	(0.84, 1.35)	0.612
20 001–30 000	0.98	(0.76, 1.26)	0.875	1.09	(0.85, 1.41)	0.490
30 001–45 000	0.56	(0.39, 0.82)	0.003	0.70	(0.47, 1.03)	0.072
> 45 000	0.28	(0.15, 0.54)	0.0001	0.33	(0.17, 1.02)	0.061
Urbanization level (Ref. rural)						
Urban	0.91	(0.73, 1.13)	0.3857	1.06	(0.85, 1.33)	0.600
Cigarette smoking habit (Ref. nonsmoker)	1.36	(1.14, 1.60)	0.0002	1.21	(1.12, 1.59)	0.011
Alcohol‐related diseases (Ref. no alcohol‐related diseases)	7.11	(4.9, 10.33)	<0.001	6.42	(4.34, 9.47)	<0.001
Comorbidities						
Diabetes	1.26	(1, 1.59)	0.0475	1.23	(0.85, 1.95)	0.124
Hypertension	1.25	(1.03, 1.53)	0.0278	1.00	(0.79, 1.27)	0.997
Chronic obstructive pulmonary disease	1.25	(0.96, 1.64)	0.0992	0.88	(0.66, 1.18)	0.399
Gastroesophageal reflux disease	1.42	(0.76, 2.68)	0.2736	1.19	(0.63, 2.27)	0.588
Barrett's esophagus	0.79	(0.2, 3.18)	0.7442	0.49	(0.12, 2)	0.321
Obesity	1.09	(0.41, 2.91)	0.8687	1.55	(0.58, 4.17)	0.386
Achalasia	1.00	(0.41, 2.46)	0.995	1.00	(0.41, 2.45)	0.994
Tylosis (Howel–Evans syndrome)	1.08	(0.27, 4.32)	0.9168	0.81	(0.2, 3.24)	0.760
Plummer–Vinson syndrome	1.56	(0.72, 3.35)	0.258	1.57	(0.73, 3.38)	0.251
Medication use						
Aspirin	1.07	(0.87, 1.3)	0.5271	0.75	(0.60, 1.04)	0.114
Metformin	0.92	(0.73, 1.16)	0.4605	0.73	(0.53, 1.09)	0.144
PPI	1.14	(0.91, 1.23)	0.5310	1.12	(0.95, 1.79)	0.397
CCI (Ref. 0)						
CCI ≥1	1.21	(0.98, 1.5)	0.0778	1.00	(0.78, 1.26)	0.964

Abbreviations: aHR, adjusted hazard ratio; CCI, Charlson Comorbidity Index; CI, confidence interval; ESCC, esophageal squamous cell carcinoma; HR, hazard ratio; NTD, New Taiwan dollars; PPI, proton pump inhibitor; Ref., reference group.

^a^ All covariates in Table 2 are adjusted.

### IRs, IRRs, and aHRs for ESCC among statin users and nonusers

Table 3 presents the relationship between statin use and ESCC development in our cohort. The incidence rate of ESCC was significantly lower in statin users than in nonusers (2.03 vs. 3.02 per 100 000 person‐years). Statin users had a lower incidence rate ratio of 0.66 for ESCC (95% CI: 0.43–0.85) relative to nonusers. After adjustments were made for age, sex, income level, urbanization level, cigarette smoking habit, alcohol‐related diseases, comorbidities, medication use, and CCI score, the risk of ESCC was significantly lower among statin users than among nonusers (aHR, 0.68; 95% CI: 0.51–0.91).

**TABLE 3:** Risk of esophageal squamous cell carcinoma in patients with betel nut chewing habits: estimated incidence rate ratios and adjusted hazard ratios.

	Events	Person‐years	IR, 100000 person‐year (10 000 person‐year)	IRR	95% CI for IRR	aHR^a^	95% CI for HR	*p*
Statin users								
Nonusers	314	709304.4	3.02	Ref.		Ref.		
Users	121	729776.2	2.03	0.66	(0.43, 0.85)	0.68	(0.51, 0.91)	0.010
Cumulative dose of statins (cDDD)								
Nonusers	314	709304.4	3.02	Ref.		Ref.		
Statin user dose, Q1 (28–182)	44	147837.4	3.01	0.98	(0.76, 2.00)	0.92	(0.54, 2.34)	0.760
Statin user dose, Q2 (183–488)	31	149820.1	2.94	0.91	(0.87, 1.58)	0.89	(0.42, 1.88)	0.761
Statin user dose, Q3 (489–1043)	26	180076.0	2.44	0.71	(0.59, 0.82)	0.66	(0.31, 1.40)	0.024
Statin user dose, Q4 (>1044)	20	252042.7	2.22	0.64	(0.55, 0.89)	0.64	(0.30, 1.36)	0.026

Abbreviations: aHR, adjusted hazard ratio; CI, confidence interval; HR, hazard ratio; CI, confidence interval; ESCC, esophageal squamous cell carcinoma; cDDD, cumulative defined daily dose; IQR, interquartile range; Q, Quartile; IR, incidence rate; IRR, incidence rate ratio; Ref., reference.

^a^ All covariates in Table 2 are adjusted.

**FIGURE 1:**
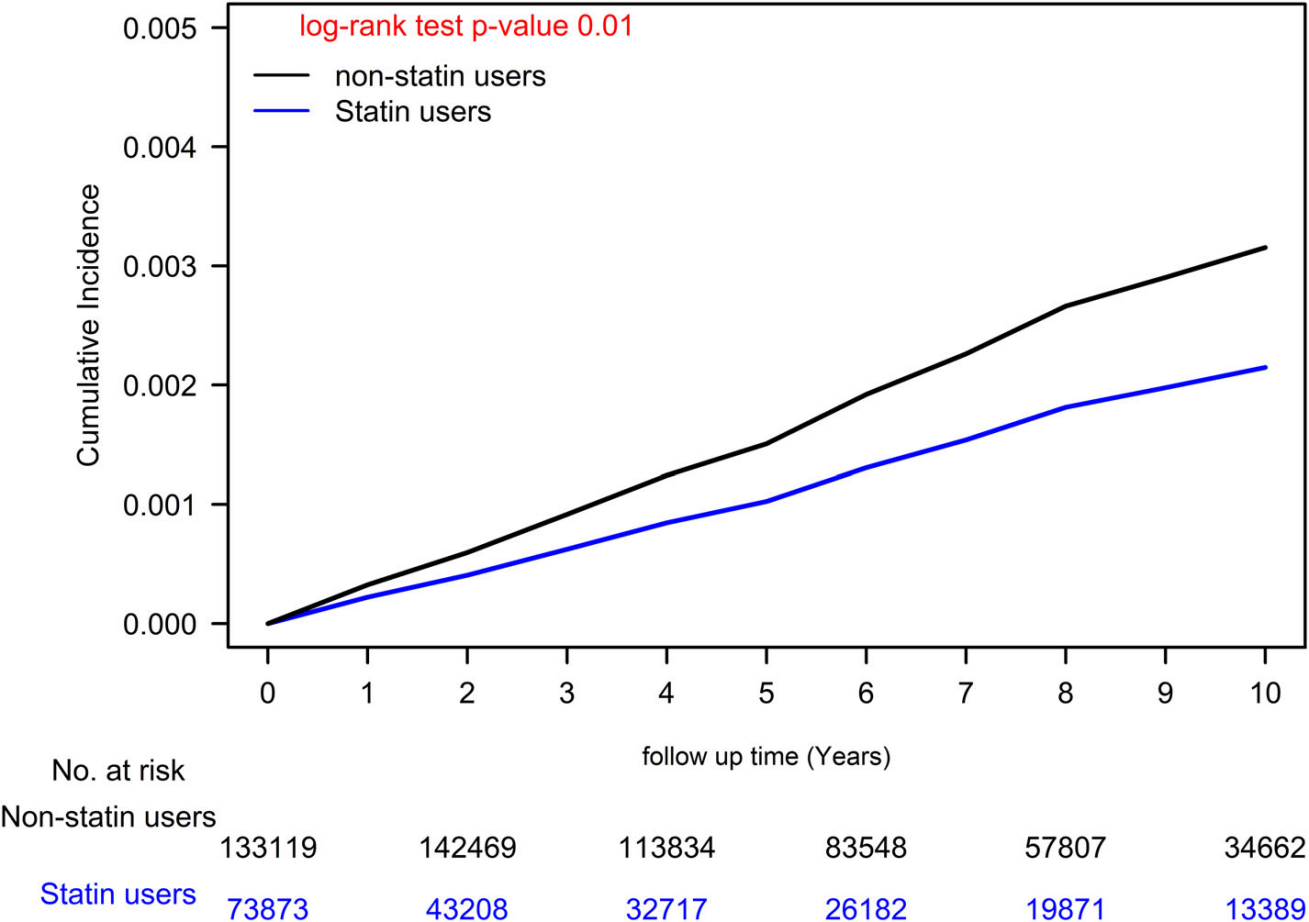
Time‐varying Cox model analysis results pertaining to cumulative incidence of esophageal squamous cell carcinoma in patients with betel nut chewing habits who are statin users or nonusers.

**FIGURE 2:**
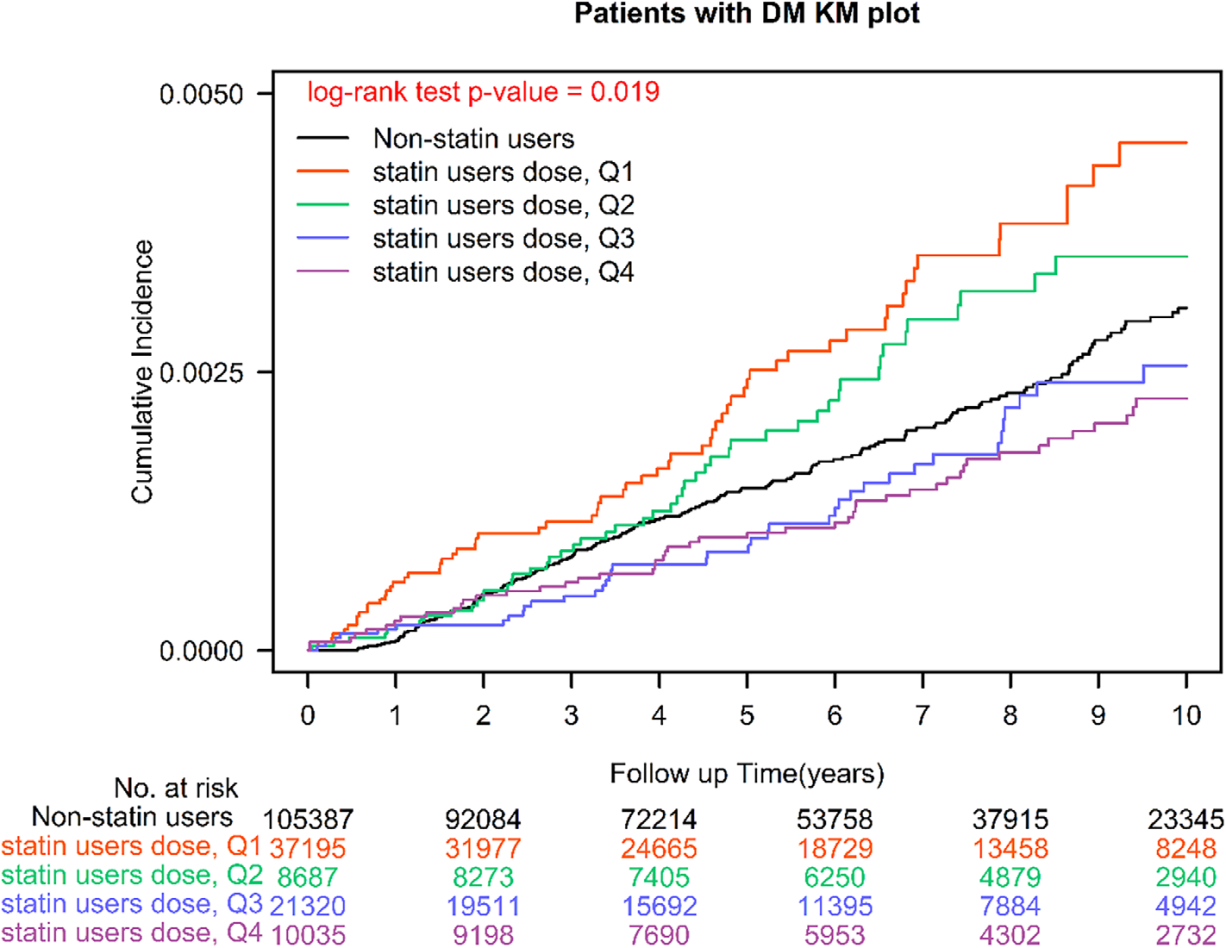
Kaplan–Meier analysis results pertaining to cumulative incidence of esophageal squamous cell carcinoma in patients with betel nut chewing habits (cumulative defined daily doses of statins).

We also observed a dose–response relationship between statin use and ESCC risk, compared with statin nonuse (<28 cDDDs), the aHRs for statin use at 28–182 cDDDs, 183–488 cDDDs, 489–1043 cDDDs, and >1043 cDDDs were 0.92, 0.89, 0.66, and 0.64, respectively. The *p*‐value for the dose–response relationship trend was <0.001. The Kaplan–Meier analysis revealed that the ESCC risk was higher in statin users than in statin nonusers (Figure 1; log‐rank test, *p* = 0.001). A similar trend was observed after the patients were stratified by statin cDDD (Figure 2; log‐rank test, *p* = 0.001).

## DISCUSSION

The chewing of areca nuts or betel quid (a mixture of areca nuts wrapped in betel leaves) is a widespread habit in numerous regions of Asia, and it is strongly implicated in the development of ESCC.^6^, ^7^ The mechanism underlying the association of areca nut chewing with ESCC may involve the release of copper, which induces collagen synthesis by fibroblasts.^8^ Several studies have demonstrated that areca nut chewing is not only significantly and independently associated with an increased risk of ESCC^38^ but also associated with a younger age of diagnosis, poor chemotherapy response, poor radiotherapy response, and a shorter overall survival in patients with ESCC.^9^ Therefore, for regions with a high prevalence of betel nut chewing (e.g., Taiwan),^4^, ^39^ the safety and long‐term effects of medications that provide protection against ESCC are crucial topics that must be investigated. Few studies have reported on the IR, IRR, and aHR of ESCC among betel nut chewers. Our study is the first to report on the IR, IRR, and aHR of ESCC among a population of betel nut chewers and estimate the other risk factors of ESCC in this population. Our results reveal that old age (>50 years), being male, smoking, and having alcohol‐related diseases are independent risk factors for ESCC in betel nut chewers. Additionally, this is the first study to report that statin use has an independent protective effect against ESCC in patients with betel nut chewing habits (Tables 2 and 3 and Figure 1) and that statin use reduces the risk of ESCC incidence (i.e., dose–response relationship; Table 3 and Figure 2).

The mechanism through which statins reduce the risk of ESCC in a population with betel nut chewing habits remains unclear. Studies have suggested that statins inhibit cancer cell growth and spread, reduce proteolysis, and produces various effects (i.e., antiproliferative, antiangiogenic, proapoptotic, and immunomodulatory effects) through both statin‐dependent and independent pathways.^40^, ^41^, ^42^, ^43^, ^44^, ^45^, ^46^, ^47^ Because the underlying mechanism of ESCC may involve collagen synthesis by fibroblasts due to betel nut chewing,^8^ statins may modulate fibrosis progression through pathways other than inflammation.^48^ This phenomenon may contribute to the reduction of ESCC risk.^41^, ^42^, ^43^, ^44^, ^45^, ^46^, ^47^, ^48^ Despite the diverse mechanisms of statins, the specific mechanisms that contribute to the reduction of ESCC risk in betel nut chewers remains unclear. Our study is the first long‐term cohort study to indicate that statins are associated with a reduced ESCC risk in patients with betel nut chewing habits. However, it also highlights that the related mechanism is still unclear.

Studies have reported that statins provide protective effects against various types of esophageal cancer (e.g., adenocarcinoma). However, none have specifically focused on patients with betel nut chewing habits or on ESCC.^24^, ^25^, ^26^ We verified the association of statin use with reduced ESCC risk in patients with betel nut chewing habits. This is a major finding because patients with betel nut chewing habits have a high risk of developing ESCC and other head and neck cancers. In future studies, we will further investigate the association between statin use and the risk of head and neck cancer in patients with betel nut chewing habits.

Our study reveals that several factors are associated with an increased risk of ESCC in betel nut chewers, namely older age (>50 years), male sex, cigarette smoking, and having alcohol‐related diseases (Table 2). These results are consistent with those of other studies on the risk factors for ESCC.^49^, ^50^, ^51^, ^52^ Our study verified that the risk factors for ESCC in betel nut chewers are similar to those reported in other studies. Therefore, in addition to statin use, quitting betel nut chewing, smoking, and alcohol are key factors that can reduce ESCC incidence.

The present study has several strengths, namely its use of a large sample size of betel nut chewers and a validation cohort, its long‐term follow‐up time, and its examination of the homogenous covariates between cases and controls after PSM and the long‐term verification of medication data. However, several limitations must also be considered. First, although the National Health Insurance Administration routinely reviews patient charts to ensure the quality of the claims submitted by medical institutions, data miscoding or misclassification can still occur. Second, several unmeasured confounders related to ESCC (e.g., body mass index) were not included in our database. Third, we could not contact patients directly to confirm their statin use because their data were anonymized; thus, the nonadherence of patients to their prescribed medication regimen was unaccounted for. Our study reveals that statin use amounting to ≥28 cDDDs is a protective factor for ESCC. Specifically, we discovered that the patients who were prescribed statin at a dosage of ≥28 cDDDs had a significantly reduced risk of ESCC with a dose–response relationship. This finding indicates that using statin at a higher cDDD provides a greater protective effect against ESCC in patients with betel nut chewing habits. To verify these findings and further clarify the optimal safety dosage and duration of statin use for reducing the risk of ESCC, a large‐scale randomized controlled trial must be conducted to compare carefully selected patients who are statin users or nonusers. Finally, the laboratory and clinical data used in the present study were not readily accessible through an administrative database.

In conclusion, our study revealed that statin use amounting to ≥28 cDDDs is a protective factor for ESCC and that patients who were prescribed statin at a dosage of ≥28 cDDDs had a significantly reduced risk of ESCC with a dose–response relationship. This finding indicates that using statin at a higher cDDD provides a greater protective effect against ESCC in patients with betel nut chewing habits.

## AUTHOR CONTRIBUTIONS

Conception and design: Chih‐Lang Lin; Wan‐Ming Chen; Ben‐Chang Shia; Szu‐Yuan Wu. Collection and assembly of data: Chih‐Lang Lin. Data analysis and interpretation: Wan‐Ming Chen; Ben‐Chang Shia; Szu‐Yuan Wu. Administrative support: Szu‐Yuan Wu. Manuscript writing: Chih‐Lang Lin; Szu‐Yuan Wu. Final approval of manuscript: All authors.

## CONFLICT OF INTEREST STATEMENT

The authors have no potential conflicts of interest to declare. The data sets supporting the study conclusions are included in the manuscript.

## Supporting information


**Data S1:** Supporting Information.

## Data Availability

The data sets supporting the study conclusions are included in the manuscript. We used data from the National Health Insurance Research Database and Taiwan Cancer Registry database. The authors confirm that, for approved reasons, some access restrictions apply to the data underlying the findings. The data used in this study cannot be made available in the manuscript, the supplemental files, or in a public repository due to the Personal Information Protection Act executed by Taiwan's government, starting in 2012. Requests for data can be sent as a formal proposal to obtain approval from the ethics review committee of the appropriate governmental department in Taiwan. Specifically, links regarding contact information for which data requests may be sent to are as follows: http://nhird.nhri.org.tw/en/Data_Subsets.html#S3 and http://nhis.nhri.org.tw/point.html. Informed consent was waived because the data sets are covered under the Personal Information Protection Act. Szu‐Yuan Wu, MD, PhD had full access to all the data in the study and takes responsibility for the integrity of the data and the accuracy of the data analysis.

## References

[1] Lin CF , Wang JD , Chen PH , Chang SJ , Yang YH , Ko YC . Predictors of betel quid chewing behavior and cessation patterns in Taiwan aborigines. BMC Public Health. 2006;6:271. 10.1186/1471-2458-6-271 17081309 PMC1636638

[2] Nelson BS , Heischober B . Betel nut: a common drug used by naturalized citizens from India, Far East Asia, and the South Pacific Islands. Ann Emerg Med. 1999;34:238–43. 10.1016/s0196-0644(99)70239-8 10424931

[3] Ko YC , Huang YL , Lee CH , Chen MJ , Lin LM , Tsai CC . Betel quid chewing, cigarette smoking and alcohol consumption related to oral cancer in Taiwan. J Oral Pathol Med. 1995;24:450–3. 10.1111/j.1600-0714.1995.tb01132.x 8600280

[4] Ko YC , Chiang TA , Chang SJ , Hsieh SF . Prevalence of betel quid chewing habit in Taiwan and related sociodemographic factors. J Oral Pathol Med. 1992;21:261–4. 10.1111/j.1600-0714.1992.tb01007.x 1501158

[5] Wen CP , Tsai MK , Chung WS , Hsu HL , Chang YC , Chan HT , et al. Cancer risks from betel quid chewing beyond oral cancer: a multiple‐site carcinogen when acting with smoking. Cancer Causes Control. 2010;21:1427–35. 10.1007/s10552-010-9570-1 20458529

[6] Pickwell SM , Schimelpfening S , Palinkas LA . 'Betelmania'. Betel quid chewing by Cambodian women in the United States and its potential health effects. West J Med. 1994;160:326–30. 8023480 PMC1022421

[7] Akhtar S , Sheikh AA , Qureshi HU . Chewing areca nut, betel quid, oral snuff, cigarette smoking and the risk of oesophageal squamous‐cell carcinoma in south Asians: a multicentre case‐control study. Eur J Cancer. 2012;48:655–61. 10.1016/j.ejca.2011.06.008 21733677

[8] Trivedy C , Baldwin D , Warnakulasuriya S , Johnson N , Peters T . Copper content in Areca catechu (betel nut) products and oral submucous fibrosis. Lancet. 1997;349:1447. 10.1016/S0140-6736(97)24020-1 9164320

[9] Chen CH , Lu HI , Wang YM , Chen YH , Lo CM , Huang WT , et al. Areca nut is associated with younger age of diagnosis, poor chemoradiotherapy response, and shorter overall survival in esophageal squamous cell carcinoma. PLoS One. 2017;12:e0172752. 10.1371/journal.pone.0172752 28245263 PMC5330470

[10] Lin WC , Chang CL , Hsu HL , Yuan KS , Wu ATH , Wu SY . Three‐dimensional conformal radiotherapy‐based or intensity‐modulated radiotherapy‐based concurrent chemoradiotherapy in patients with thoracic esophageal squamous cell carcinoma. Cancers (Basel). 2019;11. 10.3390/cancers11101529 PMC6826542 31658709

[11] Yen YC , Chang JH , Lin WC , Chiou JF , Chang YC , Chang CL , et al. Effectiveness of esophagectomy in patients with thoracic esophageal squamous cell carcinoma receiving definitive radiotherapy or concurrent chemoradiotherapy through intensity‐modulated radiation therapy techniques. Cancer. 2017;123:2043–53. 10.1002/cncr.30565 28152166

[12] Lin WC , Ding YF , Hsu HL , Chang JH , Yuan KS , Wu ATH , et al. Value and application of trimodality therapy or definitive concurrent chemoradiotherapy in thoracic esophageal squamous cell carcinoma. Cancer. 2017;123:3904–15. 10.1002/cncr.30823 28608916

[13] Chang CL , Tsai HC , Lin WC , Chang JH , Hsu HL , Chow JM , et al. Dose escalation intensity‐modulated radiotherapy‐based concurrent chemoradiotherapy is effective for advanced‐stage thoracic esophageal squamous cell carcinoma. Radiother Oncol. 2017;125:73–9. 10.1016/j.radonc.2017.08.025 28923576

[14] Pinal‐Fernandez I , Casal‐Dominguez M , Mammen AL . Statins: pros and cons. Med Clin (Barc). 2018;150:398–402. 10.1016/j.medcli.2017.11.030 29292104 PMC6019636

[15] Chan KK , Oza AM , Siu LL . The statins as anticancer agents. Clin Cancer Res. 2003;9:10–9. 12538446

[16] Dulak J , Jozkowicz A . Anti‐angiogenic and anti‐inflammatory effects of statins: relevance to anti‐cancer therapy. Curr Cancer Drug Targets. 2005;5:579–94. 10.2174/156800905774932824 16375664 PMC1391922

[17] Kim DS , Kim HJ , Ahn HS . Statins and the risk of gastric, colorectal, and esophageal cancer incidence and mortality: a cohort study based on data from the Korean national health insurance claims database. J Cancer Res Clin Oncol. 2022;148:2855–65. 10.1007/s00432-022-04075-1 35660949 PMC11801058

[18] Simon TG , King LY , Zheng H , Chung RT . Statin use is associated with a reduced risk of fibrosis progression in chronic hepatitis C. J Hepatol. 2015;62:18–23. 10.1016/j.jhep.2014.08.013 25135867 PMC4272642

[19] Simon TG , Bonilla H , Yan P , Chung RT , Butt AA . Atorvastatin and fluvastatin are associated with dose‐dependent reductions in cirrhosis and hepatocellular carcinoma, among patients with hepatitis C virus: results from ERCHIVES. Hepatology. 2016;64:47–57. 10.1002/hep.28506 26891205 PMC4917438

[20] Roehlen N , Crouchet E , Baumert TF . Liver fibrosis: mechanistic concepts and therapeutic perspectives. Cells. 2020;9. 10.3390/cells9040875 PMC7226751 32260126

[21] Blanco‐Colio LM , Tunon J , Martin‐Ventura JL , Egido J . Anti‐inflammatory and immunomodulatory effects of statins. Kidney Int. 2003;63:12–23. 10.1046/j.1523-1755.2003.00744.x 12472764

[22] Koushki K , Shahbaz SK , Mashayekhi K , Sadeghi M , Zayeri ZD , Taba MY , et al. Anti‐inflammatory action of statins in cardiovascular disease: the role of inflammasome and toll‐like receptor pathways. Clin Rev Allergy Immunol. 2021;60:175–99. 10.1007/s12016-020-08791-9 32378144 PMC7985098

[23] Weitz‐Schmidt G . Statins as anti‐inflammatory agents. Trends Pharmacol Sci. 2002;23:482–6. 10.1016/s0165-6147(02)02077-1 12368073

[24] Singh S , Singh AG , Singh PP , Murad MH , Iyer PG . Statins are associated with reduced risk of esophageal cancer, particularly in patients with Barrett's esophagus: a systematic review and meta‐analysis. Clin Gastroenterol Hepatol. 2013;11:620–9. 10.1016/j.cgh.2012.12.036 23357487 PMC3660516

[25] Zhou C , Zhong X , Gao P , Wu Z , Shi J , Guo Z , et al. Statin use and its potential therapeutic role in esophageal cancer: a systematic review and meta‐analysis. Cancer Manag Res. 2019;11:5655–63. 10.2147/CMAR.S193945 31417309 PMC6592054

[26] Nguyen T , Duan Z , Naik AD , Kramer JR , El‐Serag HB . Statin use reduces risk of esophageal adenocarcinoma in US veterans with Barrett's esophagus: a nested case‐control study. Gastroenterology. 2015;149:1392–8. 10.1053/j.gastro.2015.07.009 26208896

[27] Wen CP , Tsai SP , Chung WS . A 10‐year experience with universal health insurance in Taiwan: measuring changes in health and health disparity. Ann Intern Med. 2008;148:258–67. 18283203 10.7326/0003-4819-148-4-200802190-00004

[28] Sun M , Chen WM , Wu SY , Zhang J . Dementia risk after major elective surgery based on the route of anaesthesia: a propensity score‐matched population‐based cohort study. EClinicalMedicine. 2023;55:101727. 10.1016/j.eclinm.2022.101727 36386032 PMC9641180

[29] Wu SY , Chen WM , Chen YC , Chiang MF , Lee MC , Soong RS . Effects of H1‐antihistamines on hepatocellular carcinoma risk in patients with type 2 diabetes mellitus. Diabetes Metab. 2022;49:101393. 10.1016/j.diabet.2022.101393 36170945

[30] Sun MY , Chang CL , Lu CY , Wu SY , Zhang JQ . Sarcopenia as an independent risk factor for specific cancers: a propensity score‐matched Asian population‐based cohort study. Nutrients. 2022;14. 10.3390/nu14091910 PMC9105218 35565877

[31] Sun M , Lin JA , Chang CL , Wu SY , Zhang J . Association between long‐term opioid use and cancer risk in patients with chronic pain: a propensity score‐matched cohort study. Br J Anaesth. 2022;129:84–91. 10.1016/j.bja.2022.04.014 35597621

[32] Sun M , Chang CL , Lu CY , Zhang J , Wu SY . Effect of opioids on cancer survival in patients with chronic pain: a propensity score‐matched population‐based cohort study. Br J Anaesth. 2022;128:708–17. 10.1016/j.bja.2021.12.051 35144799

[33] Huang CC , Lin CN , Chung CH , Hwang JS , Tsai ST , Wang JD . Cost‐effectiveness analysis of the oral cancer screening program in Taiwan. Oral Oncol. 2019;89:59–65. 10.1016/j.oraloncology.2018.12.011 30732960

[34] Shen YC , Hsu HC , Lin TM , Chang YS , Hu LF , Chen LF , et al. H1‐antihistamines reduce the risk of hepatocellular carcinoma in patients with hepatitis B virus, hepatitis C virus, or dual hepatitis B virus‐hepatitis C virus infection. J Clin Oncol. 2022;40:1206–19. 10.1200/JCO.21.01802 35044851 PMC8987217

[35] Zhang Z , Reinikainen J , Adeleke KA , Pieterse ME , Groothuis‐Oudshoorn CGM . Time‐varying covariates and coefficients in cox regression models. Ann Transl Med. 2018;6:121. 10.21037/atm.2018.02.12 29955581 PMC6015946

[36] Austin PC . Optimal caliper widths for propensity‐score matching when estimating differences in means and differences in proportions in observational studies. Pharm Stat. 2011;10:150–61. 10.1002/pst.433 20925139 PMC3120982

[37] Shao YJ , Chan TS , Tsai K , Wu SY . Association between proton pump inhibitors and the risk of hepatocellular carcinoma. Aliment Pharmacol Ther. 2018;48:460–8. 10.1111/apt.14835 29897132

[38] Akhtar S . Areca nut chewing and esophageal squamous‐cell carcinoma risk in Asians: a meta‐analysis of case‐control studies. Cancer Causes Control. 2013;24:257–65. 10.1007/s10552-012-0113-9 23224324

[39] Wen CP , Tsai SP , Cheng TY , Chen CJ , Levy DT , Yang HJ , et al. Uncovering the relation between betel quid chewing and cigarette smoking in Taiwan. Tob Control. 2005;14(Suppl 1):i16–22. 10.1136/tc.2004.008003 15923442 PMC1766184

[40] Chen Y , Li LB , Zhang J , Tang DP , Wei JJ , Zhuang ZH . Simvastatin, but not pravastatin, inhibits the proliferation of esophageal adenocarcinoma and squamous cell carcinoma cells: a cell‐molecular study. Lipids Health Dis. 2018;17:290. 10.1186/s12944-018-0946-7 30579354 PMC6303879

[41] Sun HY , Singh N . Antimicrobial and immunomodulatory attributes of statins: relevance in solid‐organ transplant recipients. Clin Infect Dis. 2009;48:745–55. 10.1086/597039 19193110

[42] Wu J , Wong WW , Khosravi F , Minden MD , Penn LZ . Blocking the Raf/MEK/ERK pathway sensitizes acute myelogenous leukemia cells to lovastatin‐induced apoptosis. Cancer Res. 2004;64:6461–8. 10.1158/0008-5472.CAN-04-0866 15374955

[43] Rao S , Porter DC , Chen X , Herliczek T , Lowe M , Keyomarsi K . Lovastatin‐mediated G1 arrest is through inhibition of the proteasome, independent of hydroxymethyl glutaryl‐CoA reductase. Proc Natl Acad Sci U S A. 1999;96:7797–802. 10.1073/pnas.96.14.7797 10393901 PMC22141

[44] Kuoppala J , Lamminpaa A , Pukkala E . Statins and cancer: a systematic review and meta‐analysis. Eur J Cancer. 2008;44:2122–32. 10.1016/j.ejca.2008.06.025 18707867

[45] Kalluri R , Neilson EG . Epithelial‐mesenchymal transition and its implications for fibrosis. J Clin Invest. 2003;112:1776–84. 10.1172/JCI20530 14679171 PMC297008

[46] Kisseleva T , Brenner DA . Mechanisms of fibrogenesis. Exp Biol Med (Maywood). 2008;233:109–22. 10.3181/0707-MR-190 18222966

[47] Schuppan D , Afdhal NH . Liver cirrhosis. Lancet. 2008;371:838–51. 10.1016/S0140-6736(08)60383-9 18328931 PMC2271178

[48] Kou L , Kou P , Luo G , Wei S . Progress of statin therapy in the treatment of idiopathic pulmonary fibrosis. Oxid Med Cell Longev. 2022;2022:6197219. 10.1155/2022/6197219 35345828 PMC8957418

[49] Li H , Wu H , Cao M , Yu Y , Zhou J , Zhang S , et al. Long‐term incidence rates of esophageal squamous cell carcinoma in Chinese patients with low‐grade intraepithelial neoplasia and Association of Surveillance Endoscopy with Incidence. JAMA Netw Open. 2022;5:e2247415. 10.1001/jamanetworkopen.2022.47415 36534402 PMC9856485

[50] Abnet CC , Arnold M , Wei WQ . Epidemiology of esophageal squamous cell carcinoma. Gastroenterology. 2018;154:360–73. 10.1053/j.gastro.2017.08.023 28823862 PMC5836473

[51] Yang X , Chen X , Zhuang M , Yuan Z , Nie S , Lu M , et al. Smoking and alcohol drinking in relation to the risk of esophageal squamous cell carcinoma: a population‐based case‐control study in China. Sci Rep. 2017;7:17249. 10.1038/s41598-017-17617-2 29222520 PMC5722909

[52] Gammon MD , Schoenberg JB , Ahsan H , Risch HA , Vaughan TL , Chow WH , et al. Tobacco, alcohol, and socioeconomic status and adenocarcinomas of the esophagus and gastric cardia. J Natl Cancer Inst. 1997;89:1277–84. 10.1093/jnci/89.17.1277 9293918

