# Supplementary material for: Statin use and its association with decreased risk of esophageal squamous cell carcinoma in betel nut chewers
Source: Thorac Cancer. 2023 Jul 3;14(23):2241–50. doi: 10.1111/1759-7714.15009 (PMC10423659; doi:10.1111/1759-7714.15009)
Supplement: Supplementary file 1 — Data S1: Supporting Information. [file TCA-14-2241-s001.pdf]

**Supplemental Table 1. Comparison of Characteristics between Propensity Scores Matching Postoperative Delirium and Non-Delirium Groups in propensity scores patching Older adults Receiving Hip Fracture Surgery**

| | Non-delirium | | Delirium | | ASMD |
|---|---|---|---|---|---|
| | N=6,795 | | N=6,795 | | |
| | N | % | N | % | |
| **Age** (mean ± SD), years-old | 78.01 ± 10.22 | | 78.07 ± 11.44 | | |
| Age, median (IQR, Q1, Q3), | 78.00 (70.00,78.00) | | 78.00 (70.00,78.00) | | |
| Age group, years-old | | | | | 0.0270 |
| Group 1: 65-70 | 2,110 | 31.1% | 2,054 | 30.2% | |
| Group 2: 71-75 | 1,068 | 15.7% | 1,114 | 16.4% | |
| Group 3: 76-80 | 1,514 | 22.3% | 1,482 | 21.8% | |
| Group 4: >80 | 2,103 | 31.0% | 2,145 | 31.6% | |
| **Sex** | | | | | 0.0111 |
| Female | 2,697 | 39.7% | 2,660 | 39.2% | |
| Male | 4,098 | 60.3% | 4,135 | 60.9% | |
| **Income levels (NTD)** | | | | | 0.0440 |
| Unemployment | 136 | 2.0% | 159 | 2.3% | |
| Financial dependent | 2,782 | 40.9% | 2,667 | 39.3% | |
| ≦20000 | 2,159 | 31.8% | 2,177 | 32.0% | |
| 2000-30000 | 1,094 | 16.1% | 1,119 | 16.5% | |
| 30000-45000 | 425 | 6.3% | 456 | 6.7% | |
| ＞45000 | 199 | 2.9% | 217 | 3.2% | |
| **Urbanization** | | | | | 0.0314 |
| Rural | 2,207 | 32.5% | 2,108 | 31.0% | |
| Urban | 4,588 | 67.5% | 4,687 | 69.0% | |
| **ASA physical status** | | | | | 0.0280 |

For Review Only

| | | | | | |
|---|---|---|---|---|---|
| 1 | 3,000 | 44.2% | 2,960 | 43.6% | |
| 2 | 1,028 | 15.1% | 986 | 14.5% | |
| 3 | 1,508 | 22.2% | 1,532 | 22.6% | |
| 4 | 1,259 | 18.5% | 1,317 | 19.4% | |
| **Types of anesthesia** | | | | | 0.0180 |
| General anesthesia | 6,077 | 89.4% | 6,039 | 88.9% | |
| Regional anesthesia | 718 | 10.6% | 756 | 11.1% | |
| **Duration of anesthesia** | | | | | 0.0011 |
| ≤ 2 hours | 5,833 | 85.8% | 5,830 | 85.8% | |
| >2 hours | 962 | 14.2% | 965 | 14.2% | |
| **Pre-existing comorbidity** | | | | | |
| Diabetes | 974 | 14.3% | 1,034 | 15.2% | 0.0251 |
| Hypertension | 2,008 | 29.6% | 2,115 | 31.1% | 0.0344 |
| Hyperlipidemia | 927 | 13.6% | 986 | 14.5% | 0.0250 |
| Coronary artery disease | 914 | 13.5% | 971 | 14.3% | 0.0243 |
| Stroke | 1,262 | 18.6% | 1,355 | 19.9% | 0.0348 |
| Depression | 563 | 8.3% | 589 | 8.7% | 0.0136 |
| Anxiety | 728 | 10.7% | 792 | 11.7% | 0.0301 |
| Heart failure | 328 | 4.8% | 343 | 5.1% | 0.0102 |
| Peripheral vascular disease | 399 | 5.9% | 401 | 5.9% | 0.0013 |
| COPD | 1,065 | 15.7% | 1,081 | 15.9% | 0.0066 |
| Atrial fibrillation | 116 | 1.7% | 148 | 2.2% | 0.0340 |
| Traumatic head injury | 773 | 11.4% | 775 | 11.4% | 0.0009 |
| Alcohol liver diseases | 418 | 6.2% | 421 | 6.2% | 0.0008 |
| Cognitive function decline | 429 | 6.3% | 428 | 6.3% | 0.0002 |
| Sleep Disorder | 1,390 | 20.5% | 1,393 | 20.5% | 0.0011 |

For Review Only

| | | | | | |
|---|---|---|---|---|---|
| Malnutrition | 833 | 12.3% | 836 | 12.3% | 0.0010 |
| **CCI Scores** | | | | | |
| Mean (SD) | 0.80 ± 1.47 | | 0.94 ± 1.72 | | 0.0840 |
| Median (IQR, Q1-Q3) | 0.00 (0.00,1.00) | | 0.00 (0.00,1.00) | | |
| CCI Scores | | | | | 0.0025 |
| 0 | 4,427 | 65.2% | 4,419 | 65.0% | |
| ≥1 | 2,368 | 34.9% | 2,376 | 35.0% | |
| CCI | | | | | |
| Congestive Heart Failure | 286 | 4.2% | 324 | 4.8% | 0.0270 |
| Dementia | 250 | 3.7% | 256 | 3.8% | 0.0008 |
| Chronic Pulmonary Disease | 980 | 14.4% | 890 | 13.1% | 0.0383 |
| Rheumatic Disease | 61 | 0.9% | 47 | 0.7% | 0.0236 |
| Liver Disease | 854 | 12.6% | 771 | 11.4% | 0.0376 |
| Diabetes with complications | 231 | 3.4% | 230 | 3.4% | 0.0011 |
| Hemiplegia and Paraplegia | 1 | 0.0% | 1 | 0.0% | 0.0000 |
| Renal Disease | 258 | 3.8% | 254 | 3.7% | 0.0032 |
| AIDS | 5 | 0.1% | 6 | 0.1% | 0.0071 |
| Cancer | 479 | 7.0% | 486 | 7.2% | 0.0004 |

**Abbreviations**: ASA, American Society of Anesthesiologists; CCI, Charlson Comorbidity Index; ASMD, absolute standardized mean difference; IQD, interquartile range; SD, standard deviation; COPD, Chronic Obstructive Pulmonary Disease; N, Number; NTD, New Taiwan Dollars.

For Review Only

**Supplemental Table 2. Comparison of 30-Day Postoperative Major Complications between Propensity Scores Matching Older adults Receiving Hip Fracture Surgery with and without Postoperative Delirium**

| | No Postoperative delirium | | Postoperative Delirium | | *P* Value |
|---|---|---|---|---|---|
| **30-Day postoperative complication** | **N** | **%** | **N** | **%** | |
| **30-Day Postoperative Mortality** | 141 | 2.1% | 139 | 2.1% | 0.9039 |
| **30-Day Acute Myocardial Infarction** | 38 | 0.6% | 50 | 0.7% | 0.1994 |
| **30-Day Acute Renal Failure** | 66 | 1.0% | 115 | 1.7% | 0.0002 |
| **30-Day deep-wound infection** | 38 | 0.6% | 23 | 0.3% | 0.0542 |
| **30-Day Postoperative Pneumonia** | 263 | 3.9% | 543 | 8.0% | <0.0001 |
| **30-Day Postoperative Bleeding** | 45 | 0.7% | 59 | 0.9% | 0.1682 |
| **30-Day Pulmonary Embolism** | 8 | 0.1% | 10 | 0.2% | 0.6371 |
| **30-Day Septicemia** | 333 | 4.9% | 539 | 7.9% | <0.0001 |
| **30-Day Postoperative Stroke** | 811 | 11.9% | 1,000 | 14.7% | <0.0001 |
| **Any 30-Day Major Postoperative complication** | 1,306 | 19.2% | 1,868 | 27.5% | <0.0001 |

**Abbreviations**: N, numbers.

For Review Only
